# Evaluating Insulin Delivery Systems Using Dynamic Glucose Region Plots and Risk Space Analysis

**DOI:** 10.3390/s25154788

**Published:** 2025-08-04

**Authors:** Klavs W. Hansen, Mia Christensen, Sanne Fisker, Ermina Bach, Bo M. Bibby

**Affiliations:** 1 Department of Clinical Medicine, Aarhus University, Palle Juul-Jensens Boulevard 82, 8200 Aarhus, Denmark; 2 Medical Diagnostic Center, Silkeborg Regional Hospital, Falkevej 1, 8600 Silkeborg, Denmark; 3 Steno Diabetes Center Aarhus, Aarhus University Hospital, Palle Juul Jensens Boulevard 11, 8200 Aarhus, Denmark; sanne.fisker@auh.rm.dk (S.F.); ermina.bach@midt.rm.dk (E.B.); 4 Medical Diagnostic Center, Viborg Regional Hospital, Heibergs Alle 5, 8800 Viborg, Denmark; 5 Section for Biostatistics, Department of Public Health, Aarhus University, Bartholins Allé 2, 8000 Aarhus, Denmark; bibby@biostat.au.dk

**Keywords:** type 1 diabetes, continuous glucose monitoring, glucose rate of change, dynamic glucose region plots, risk space analysis, automated insulin delivery

## Abstract

**Highlights:**

**What are the main findings?**

1. Our results reveal a clinically relevant higher fraction of (RoC, glucose) values in the optimal risk space with AID system A compared to system B.
2. The risk of glucose declining from the target range to below target range was lower in persons using AID systems than in persons without an integrated CGM system.

**What is the implication of the main finding?**

1. Risk space analysis of dynamic glucose region plots is a novel strategy that contributes to the real-world evaluation of different systems for insulin delivery.

**Abstract:**

Simultaneous values of glucose rate of change (RoC) and glucose can be presented in a dynamic glucose region plot, and risk spaces can be specified for (RoC, glucose) values expected to remain in the target range (glucose 3.9–10.0 mmol/L) or leave or return to the target range within the next 30 min. We downloaded continuous glucose monitoring (CGM) data for 60 days from persons with type 1 diabetes using two different systems for automated insulin delivery (AID), A (n = 65) or B (n = 85). The relative distribution of (RoC, glucose) values in risk spaces was compared. The fraction of all (RoC, glucose) values anticipated to remain in the target range in the next 30 min was higher with system A (62.5%) than with system B (56.8%) (difference 5.7, 95% CI (2.2–9.2%), *p* = 0.002). The fraction of (RoC, glucose) values in the target range with a risk of progressing to the above range (glucose > 10.0 mmol/L) was slightly lower in system A than in B (difference −1.1 (95% CI: −1.8–−0.5%, *p* < 0.001). Dynamic glucose region plots and the concept of risk spaces are novel strategies to obtain insight into glucose homeostasis and to demonstrate clinically relevant differences comparing two AID systems.

## 1. Introduction

Advanced hybrid closed-loop systems for insulin delivery have the potential to achieve near-normal glucose levels through features such as predictive low-glucose suspension, automated adjustment of insulin infusion rates, and supplementary correction boluses [1,2]. Although user-initiated bolusing for meals is still required, precise carbohydrate counting is no longer of paramount importance [3,4]. The term “automated insulin delivery” (AID) has been used for these systems [5]. Fundamental differences exist between the different algorithms, but they all combine information about actual glucose values from continuous glucose monitoring (CGM) and the speed and direction of glucose change [6]. The relation between glucose values and glucose rate of change (RoC) is a key factor in maintaining near-normal glucose levels. It has, for at least two decades, been an essential input for glucose prediction analyses and the development of algorithms for AID, which often has been tested in an in silico model before clinical testing [7]. According to Guerra et al., points representing simultaneous RoC and glucose values can be mapped to dynamic risk spaces with a high or low probability of maintaining glucose within the target range (3.9–10.0 mmol/L) [8]. This approach has previously been used by Eichenlaub et al. to determine the minimum percentage of comparator glucose values within specific risk spaces needed to evaluate the performance of CGM systems [9]. The resulting graphical presentation was designated a dynamic glucose region plot [8]. The comprehensive bioengineering work leading to the development of AID algorithms and alert systems for hypo- and hyperglycemia has often been presented in patents and patent applications [10] or engineering literature [11,12]. The design of closed-loop controllers is usually tested with the UVA/Padova simulator, and outcomes can be evaluated from control-variability grid analysis [13]. Recent work by Montaser et al. also supports the value of CGM data dynamics in clinical stratification and prediction, proposing novel glucose-derived markers for diabetes detection and progression [14].

This study aims to introduce dynamic glucose region plots and a simple strategy to evaluate the performance of AID systems and compare them with insulin delivery via multiple daily insulin injections (MDI) or continuous subcutaneous insulin infusion (CSII) combined with unintegrated intermittently scanned CGM (isCGM) as a reference.

## 2. Methods

We have downloaded CGM raw data for 60 days for 160 persons with type 1 diabetes using the AID systems Tandem Control IQ (CoIQ) or Minimed 780G (MM780G) in a non-randomized observational study [15]. Of these, we omitted 12 persons with an active CGM time < 70%. The comparator population is CGM data from 90 days for 159 persons with type 1 diabetes and intermittently scanned CGM (isCGM) and active CGM time > 70% [16]. Of these, we omitted 11 persons who switched to AID and are contained in the AID population. The final study population was 148 persons with AID (CoIQ, 83 persons; MM780G, 65 persons) and 148 persons with isCGM (MDI, 131 persons; CSII, 17 persons).

Glucose data were sampled every 5 min by the Dexcom G6 sensor (CoIQ) or Guardian 3 or 4 sensors (MM780G) and were downloaded from the Glooko (CoIQ) or Carelink software platform (MM780G). The isCGM group used the Abbott Freestyle Libre (version 1) sensor, which provided imported glucose readings every 15 min in addition to the values obtained when the user scanned the sensor with a receiver unit. These data were downloaded from the Glooko platform. We calculated the average of imported and scanned glucose values (if any) for each 15 min.

Active CGM time (%) was calculated as the number of 5- or 15-min periods with an available glucose value, divided by the total number of periods in 60 or 90 days, respectively, multiplied by 100.

A simultaneous rate of change (RoC) and glucose point was defined as RoC for the period t = 0 to t = 15 min in combination with glucose for t = 15 min. The RoC unit was glucose mmol/l/15 min. For the AID group, RoC was calculated as the slope of the regression line for glucose at t = 0, t = 5, t = 10, and t = 15 min, as recommended [17]. For the isCGM group, RoC was calculated from the difference between calculated glucose values with 15-min intervals. For comparison with the isCGM group, we also calculated RoC in the AID group at 15-min intervals, disregarding intermediate values.

Glucose ranges were defined according to the international consensus guidelines as follows [18]: glucose above the target range (>10 mmol/L); in target range (3.9–10.0 mmol/L); and below range (<3.9 mmol/L).

The following dynamic spaces for (RoC, glucose) points were defined:

A: Glucose is in the target range and will increase above the target range if RoC is unchanged for the next 30 min.

B: Glucose is above the target range and will decrease to the target range if RoC is unchanged for the next 30 min.

C: Glucose is in the target range and will decrease to below the target range if RoC is unchanged for the next 30 min.

D: Glucose is below the target range, and RoC is negative or 0.

E: Glucose is below the target range, and RoC is positive.

O: Glucose is in the target range and will be maintained in this range unchanged for the next 30 min.

The discrimination line for A is given by: Glucose (mmol/L) + 2RoC (mmol/L/15 min) = 10 mmol/L corresponding to glucose = −2RoC + 10.

**Figure 1:**
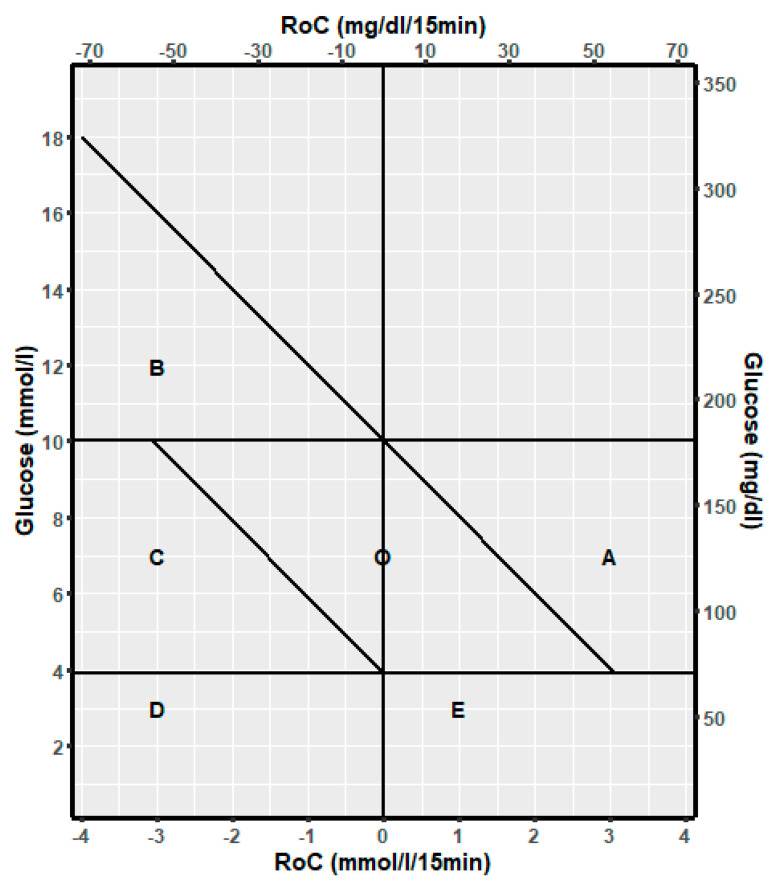
Template to describe the relation of glucose dynamic risk spaces A, B, C, D, E, and O to the spaces for glucose above the target range, in the target range, and below the target range.

Accordingly, the line for B is Glucose = −2RoC + 10, and the line for C is Glucose = −2Roc + 3.9. The risk spaces are illustrated in Figure 1.

The lowest glucose values that can be measured are 2.2 mmol/L (Dexcom G6 and Libre) or 2.8 mmol/L (Guardian). This gives a natural constraint on (RoC, glucose) points for the combination of positive RoC values and low glucose values at t = 15 min since glucose at t = 0 min cannot be below the lower detection limit. The line identifying this constraint is given by: 2.2 (or 2.8) = Glucose − RoC corresponding to: Glucose = RoC + 2.2 (or 2.8). The highest glucose value that can be measured is 22.2 mmol/L (Dexcom, Guardian) or 27.8 mmol/L (Libre). This gives a natural constraint on (RoC, glucose) points for the combination of negative RoC values and high glucose values at t = 15 min since glucose at t = 0 min cannot be higher than the detection limit. The line identifying this constraint is given by: 22.2 (or 27.8) = Glucose − RoC corresponding to: Glucose = RoC +22.2 (or 27.8).

We present the distribution of (RoC, glucose) points as the fraction of the total number of points and the fraction of the points within the relevant glucose ranges. As an example, the fraction of (RoC, glucose) points in the dynamic glucose space A is presented as the number of points in A divided by the total numbers: nA/ntotal (%) and as nA divided by the number of points in target range: nA/n target range (%).

It follows that the fractions of (RoC, glucose) points in A, C, and E should be as low as possible and the fraction in O should be as high as possible. The (RoC, glucose) points in B should be as high a fraction as possible of all points above the target range.

The dynamic glucose region plot is presented as a heat map illustrating the density of (RoC, glucose) points and a 3D plot with the density at the z axis.

## 3. Statistical Analysis

The distribution of (RoC, glucose) values within target ranges and dynamic risk spaces was compared using a generalized linear model with an identity link function, accounting for clustering by person ID. The mean risk difference and 95% confidence intervals (CIs) are presented. Heatmaps (two- and three-dimensional) were derived from the R-package ggpointdensity (version 0.2.0.) installed from Github. We present the relative density in the heatmaps as the number of (RoC, glucose) values in each of 100 × 100 squares divided by the total number.

## 4. Results

**Figure 2:**
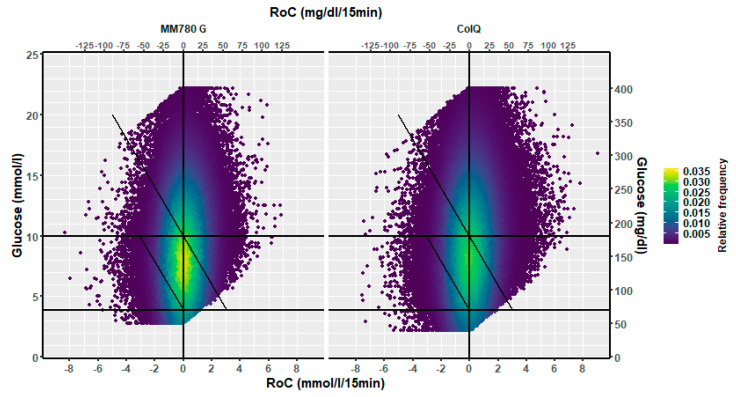
A two-dimensional heatmap of the distribution of (RoC, glucose) pairs in glucose dynamic risk spaces for (**left**) users of the MM780G (351,591 pairs) and (**right**) users of CoIQ system (461,943 pairs).

**Figure 3:**
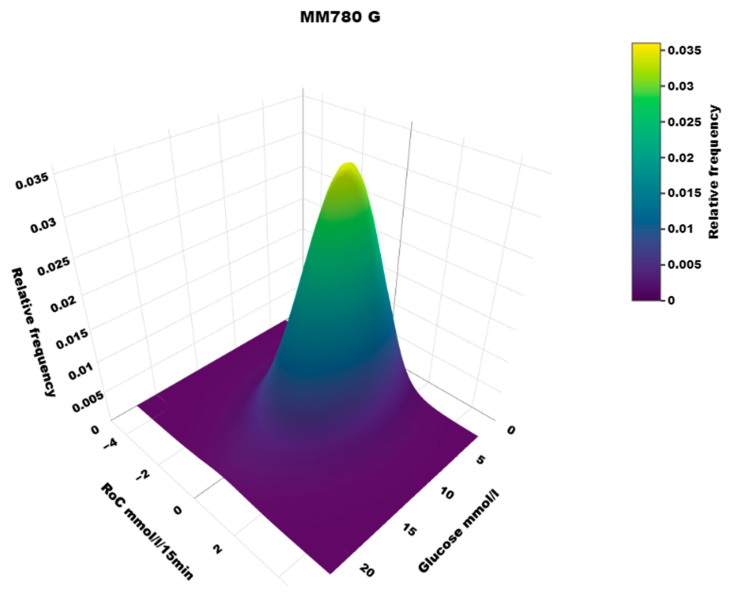
A three-dimensional heatmap of the distribution of (RoC, glucose) pairs in the MM780G system users.

**Figure 4:**
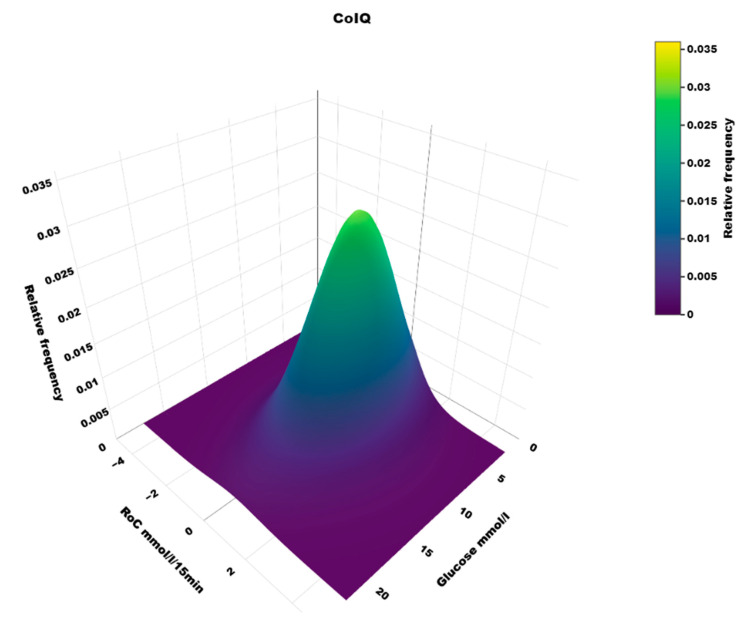
A three-dimensional heatmap of the distribution of (RoC, glucose) pairs in users of the CoIQ system.

The distribution of RoC in the dynamic glucose region plot for the two AID systems based on the slope of the regression line is shown in Table 1. The two-dimensional heatmaps are shown in Figure 2; the three-dimensional heatmaps are shown in Figure 3 (MM780G) and Figure 4 (CoIQ). The natural constraints on (RoC, glucose) values are illustrated by the sharp linear demarcation in the plots’ lower right and upper left regions of the dynamic glucose region (Figure 2). The fraction of glucose values in the target range was higher in MM780G than in the CoIQ system (difference 5.0, CI: 1.3–8.8, *p* = 0.009). The fraction of all (RoC, glucose) values in the optimal space O (nO/ntotal) was higher for MM780G than for CoIQ users (difference 5.7% (CI: 2.2–9.2%, *p* = 0.002), as was the fraction values in space O relative to the total numbers in target range (nO/nITR) (difference 2.0, CI: 0.8–3.2%, *p* = 0.002), The fraction of values in target range and the risk space A was slightly lower in the MM780G system (difference −1.1, CI: −1.8–−0.5%, *p* < 0.001). Otherwise, only minor differences were observed in the distribution of (RoC, glucose) values between the two AID groups.

**Figure 5:**
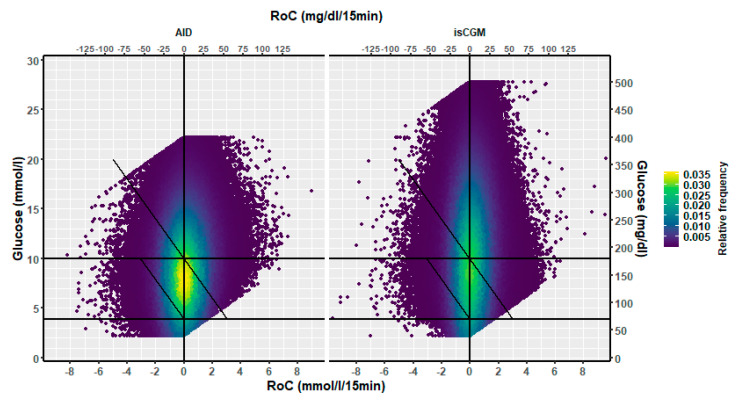
A two-dimensional heatmap of the distribution of (RoC, glucose) pairs in glucose dynamic risk spaces for users of (**left**) the AID pumps (853,534 values) and (**right**) users of isCGM (1,181,627 values).

**Figure 6:**
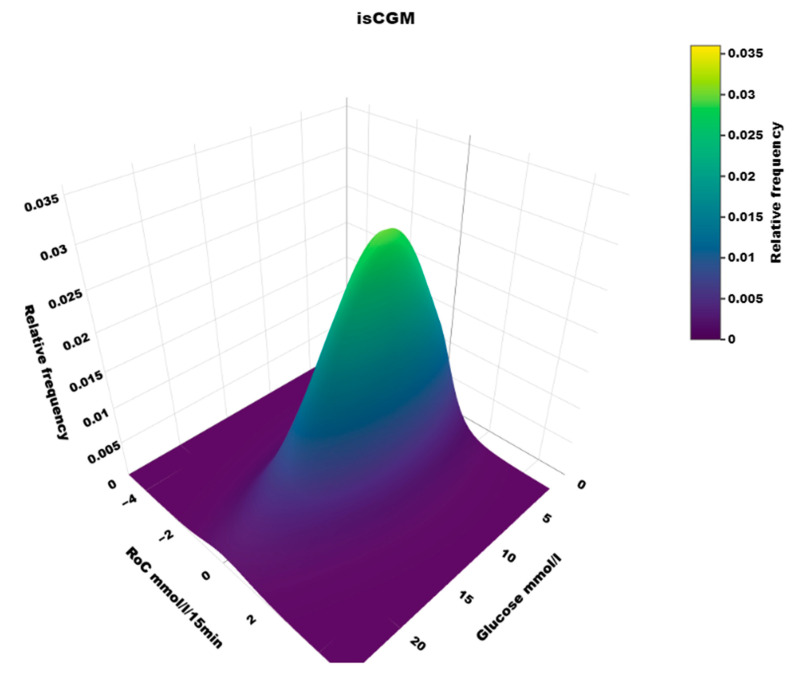
A three-dimensional heatmap of the distribution of (Roc, glucose) values in users of isCGM.

In the isCGM group, the number of scanned values was 13% of those imported with 15-min intervals. The dynamic glucose region plot for isCGM and the AID group is shown in Figure 5, and the distribution in risk spaces is shown in Table 2. The three-dimensional heatmap for the isCGM group is shown in Figure 6. The AID group had a higher fraction of (RoC, glucose) values in space B (difference 5.0, CI: 4.3–5.7%, *p* < 0.00001) and a lower fraction in space C (difference 1.5, CI: −2.2–−0.9%, *p* < 0.00001) than isCGM group.

## 5. Discussion

We present the concept of risk spaces derived from dynamic glucose region plots. To illustrate this approach, we evaluate data from persons using two different AID systems and those using unintegrated CGM.

### 5.1. Heatmaps

To our knowledge, the three-dimensional heatmap of (RoC, glucose) values has not been described previously. There is a general “orca dorsal fin” appearance of these figures, irrespective of the categories of insulin delivery. The highest density of (RoC, glucose) is uniformly corresponding to a glucose concentration of about 7–8 mmol/L and a glucose rate of change of 0. The density of values with glucose rate of change lower than −2 or higher than +2 mol/L/15 min is very low. In accordance, we have previously defined a rapid change of glucose as a rate numerically higher than 1.5 mmol/L/15 min [19]. For a clinical evaluation of heatmaps at a glance, we recommend focusing on the proportion of values with glucose > 10 mmol/L and positive RoC (hyperglycemia with increasing glucose) and glucose < 3.9 mmol/L and negative RoC (hypoglycemia with declining glucose), as these spaces represent particularly unwanted (RoC, glucose) combinations.

### 5.2. Comparison of Two AID Systems

Only minor differences in the distribution of (RoC, glucose) values were found between the two AID systems. The MM780G system had a slightly lower fraction of (RoC, glucose) values in the target range within the risk space A for increasing to above the target range within 30 min than the CoIQ system. This can contribute to the higher fraction of all glucose values in the above target range. Both AID systems deliver autobasal boluses every 5 min. The ability of the MM780G system to deliver autocorrection boluses every 5 min may be more effective in dampening rising glucose levels than the CoIQ system, which administers correction boluses at 60-min intervals. The optimal space O in the dynamic glucose region plot is a parallelogram that depicts glucose values expected to be maintained in the target range for the next 30 min. The fraction of values in space O relative to the total numbers in the target range was higher in MM780G than in CoIQ users, which may indicate a more favourable algorithm.

### 5.3. AID vs. isCGM

In the AID group, we found a higher fraction of values above the target range that fell in space B—indicative of glucose levels expected to decline into the target range within 30 min—than in the isCGM group. This difference may be attributed to the AID group’s increased basal and autocorrection boluses. The fraction of (RoC, glucose) values in the target range that carried a risk of decreasing to below the target range within 30 min (space C) was slightly lower in the AID group than in the isCGM group; suspension of insulin infusion before low glucose can contribute to this finding. The proportion of values in the target range with a risk of increasing above this range (space A) within 30 min was comparable between the AID and the isCGM groups, despite the potential for autocorrection boluses in the AID groups. This finding may be explained by carbohydrate intake in the AID group during hypoglycaemia, which may not be adequately counterbalanced when insulin infusion is temporarily suspended. This is in line with the observations of a higher frequency of rebound hyperglycaemia in persons with AID [20].

### 5.4. Impact of Using Sensors with Different Performance

Sensor-derived glycaemic metrics like time in ranges and mean glucose can be difficult to compare if obtained from different sensors [21,22,23] because of varying sensor tuning. The fractions of (RoC, glucose) pairs within a risk space, relative to the total number of pairs in TIR (ex. nA/nTIR, nC/nTIR, and nO/nITR) or TAR (nB/nTAR), are independent of sensor performances and are likely to reflect the actual capacity of the AID algorithm to maintain glucose homeostasis.

The higher fraction of glucose values in the target range and lower values above the target range in users of MM780G does not necessarily imply better glycemic control if the sensor connected to MM780G is calibrated to a lower glucose value than Dexcom G6, as is the case for the successors Simplera versus Dexcom G7 [24,25]. The consequences of different sensor calibrating are very important, as recently reported from the Ulm group in Germany, who have compared CGM results from people wearing three different sensors simultaneously. The impact of different sensors relates not only to the level of sensor glucose but also the ability to respond to induced glucose excursions [24,25].

However, the AID algorithms per se can still be evaluated in their own right from sensor data, as recently discussed [26].

### 5.5. Limitations and Strengths

The choice of AID system was not randomized, which is a limitation of our study. Future studies comparing AID systems should be randomized to avoid possible confounders related to the health care providers who individually recommend AID systems [27]. We chose a glucose predictive period of 30 min to follow the work of Eichenlaub [17], but it can be argued that a predictive period of 15 or 60 min could alter the results. We cannot exclude that the longer sampling period for isCGM introduces additional variation compared to the equal sampling period for the two AID systems. Furthermore, we do not consider the effect of age. It is a strength that we downloaded glucose raw data over a long period, producing between 350,000 and 450,000 (RoC, glucose) values for each AID system and more than 1 million values for the isCGM group. We used the same data processing independent of the software platforms for the two AID systems.

### 5.6. Static vs. Dynamic Risk Evaluation

The idea of changing from static risk scores to dynamic risk scores visualized in a (RoC, glucose) diagram is well described [8,28]. It relates to continuous error grid analysis refined to the combined assessment of rate-error grid and point-error grid zones in one matrix [29,30]. This aligns with recent findings by Montaser et al., who used CGM-derived dynamic features to define new markers for disease progression, further emphasizing the clinical value of time-series glucose analysis [14]. We suggest a simplified approach to evaluate distribution in risk spaces based on the download of the CGM time series and without the application of advanced or patented prediction analysis.

## 6. Conclusions

Dynamic glucose region plots with identified risk spaces provide insights into the performance of insulin delivery systems and can be an inspiration for real-world evaluation of different AID algorithms.

## Figures and Tables

**Table 1:** Distribution of 60 days of (RoC, glucose) values from MM780 and CoIQ pump users for time in ranges and dynamic risk spaces. RoC was calculated as the slope of the regression line between glucose at t = 0, t = 5, t = 10, and t = 15 min.

	MM780G (n = 65 Persons 351,591 RoC, Glucose Values)	CoIQ (n = 83 Persons 461,943 RoC, Glucose Values)	Mean Risk Difference (95% CI)	*p*-Value
Above target range (ATR) (%)	24.6	29.6	−5.0 (−8.9–−1.2)	0.011
In target range (ITR) (%)	73.9	68.8	5.0 (1.3–8.8)	0.009
Below target range (BTR) (%)	1.5	1.5	0.0 (−0.5–0.5)	0.98
Risk space A (%)	6.9	7.23	−0.3 (−0.7–0.1)	0.14
Risk space B (%)	3.7	4.5	−0.8 (−1.3–−0.3)	0.0013
Risk space C (%)	4.5	4.8	−0.3 (−0.9–0.3)	0.36
Risk space D (%)	1.2	1.2	−0.0 (−0.4–0.3)	0.94
Risk space E (%)	0.4	0.3	0.0 (−0.1–0.1)	0.75
Risk space O (%)	62.5	56.8	5.7 (2.2–9.2)	0.002
A/ITR (%)	9.4	10.5	−1.1 (−1.8–−0.5)	<0.001
B/ATR (%)	14.9	15.1	−0.1 (−1.0–0.7)	0.74
C/ITR (%)	6.0	6.9	−0.9 (−1.7–−0.0)	0.04
D/BTR (%)	76.7	77.9	−1.2 (−3.1–0.8)	0.24
E/BTR (%)	23.3	22.1	−1.2 (−0.8–3.1)	0.24
O/ITR (%)	84.6	82.6	2.0 (0.8–3.2)	0.002

**Table 2:** Distribution of 60 days of (RoC, glucose) values from AID pumps and 90 days (RoC, glucose) pairs from users of isCGM for time in ranges and dynamic risk spaces. RoC was calculated as the slope of the line between glucose at t = 0 and t = 15 min.

	AID Pumps (148 Persons, 813,534 RoC, Glucose Values)	isCGM (148 Persons, 1,189,627 RoC, Glucose Values)	Mean Risk Difference (95%CI)	*p*-Value
Above target range (ATR) (%)	27.4	41.3	−13.9 (−17.2–−10.6)	<0.0001
In target range (ITR) (%)	71.0	53.3	17.8 (14.7–20.9)	<0.0001
Below target range (BTR) (%)	1.5	5.4	−3.9 (−4.6–−3.1)	<0.0001
Risk space A (%)	7.0	5.4	1.6 (1.3–1.9)	<0.0001
Risk space B (%)	4.1	4.1	−0.02 (−0.3–0.3)	0.91
Risk space C (%)	4.6	4.3	0.3 (−0.1–0.8)	0.14
Risk space D (%)	1.2	4.2	−3.0 (−3.6–−2.4)	<0.0001
Risk space E (%)	0.3	1.2	−0.9 (−1.1–−0.7)	<0.0001
Risk space O (%)	59.4	43.6	15.8 (13.0–18.6)	<0.0001
A/ITR (%)	9.9	10.2	−0.3 (−0.8–0.2)	<0.0001
B/ATR (%)	15.0	10.0	5.0 (4.3–5.7)	<0.0001
C/ITR (%)	6.5	8.0	−1.5 (−2.2–−0.9)	<0.0001
D/BTR (%)	78.5	77.4	1.0 (−0.2–2.3)	0.10
E/BTR (%)	21.5	22.6	−1.0 (−2.3–0.2)	0.10
O/ITR (%)	83.6	81.8	1.8 (0.9–2.7)	<0.0001

## Data Availability

The data presented in this study are available on reasonable request from the corresponding author.
